# Laboratory evaluation of the efficacy of deltamethrin-laced attractive toxic sugar bait formulation on *Anopheles stephensi*

**DOI:** 10.1186/s12936-023-04524-3

**Published:** 2023-03-11

**Authors:** Sarita Kumar, Aarti Sharma, Roopa Rani Samal, Manoj Kumar, Vaishali Verma, Ravinder Kumar Sagar, Shri Pati Singh, Kamaraju Raghavendra

**Affiliations:** 1 Department of Zoology, Acharya Narendra Dev College, University of Delhi, Kalkaji, New Delhi, 110 019 India; 2 ICMR-National Institute of Malaria Research, Sector 8, Dwarka, New Delhi, 110 077 India; 3 Present Address: H. No. 28 B, Block ED, Pitampura, Delhi, 110 088 India

**Keywords:** *Anopheles stephensi*, Attractive toxic sugar bait (ATSB), Cage bioassay, Deltamethrin, Fruit juice

## Abstract

**Background:**

Attractive toxic sugar bait (ATSB) is a promising “attract and kill”-based approach for mosquito control. It is a combination of flower nectar/fruit juice to attract the mosquitoes, sugar solution to stimulate feeding, and a toxin to kill them. Selecting an effective attractant and optimizing concentration of toxicant is significant in the formulation of ATSB.

**Methods:**

Current study formulated an ATSB using fruit juice, sugar and deltamethrin, a synthetic pyrethroid. It was evaluated against two laboratory strains of *Anopheles stephensi*. Initial studies evaluated comparative attractiveness of nine different fruit juices to *An*.* stephensi* adults. Nine ASBs were prepared by adding fermented juices of plum, guava, sweet lemon, orange, mango, pineapple, muskmelon, papaya, and watermelon with 10% sucrose solution (w/v) in 1:1 ratio. Cage bioassays were conducted to assess relative attraction potential of ASBs based on the number of mosquito landings on each and the most effective ASB was identified. Ten ATSBs were prepared by adding the identified ASB with different deltamethrin concentrations (0.015625–8.0 mg/10 mL) in 1:9 ratio. Each ATSB was assessed for the toxic potential against both the strains of *An*.* stephensi*. The data was statistically analysed using PASW (SPSS) software 19.0 program.

**Results:**

The cage bioassays with nine ASBs revealed higher efficacy (*p* < 0.05) of Guava juice-ASB > Plum juice-ASB > Mango juice-ASB in comparison to rest of the six ASB’s. The bioassay with these three ASB’s ascertained the highest attractancy potential of guava juice-ASB against both the strains of *An*.* stephensi*. The ATSB formulations resulted in 5.1–97.9% mortality in Sonepat (NIMR strain) with calculated LC_30_, LC_50,_ and LC_90_ values of 0.17 mg deltamethrin/10 mL, 0.61 mg deltamethrin/10 mL, and 13.84 mg deltamethrin/10 mL ATSB, respectively. Whereas, 6.12–86.12% mortality was recorded in the GVD-Delhi (AND strain) with calculated LC_30_, LC_50_, and LC_90_ values of 0.25 mg deltamethrin/10 mL, 0.73 mg deltamethrin/10 mL and 10.22 mg deltamethrin/10 mL ATSB, respectively.

**Conclusion:**

The ATSB formulated with guava juice-ASB and deltamethrin (0.0015625–0.8%) in 9:1 ratio showed promising results against two laboratory strains of *An*.* stephensi*. Field assessment of these formulations is being conducted to estimate their feasibility for use in mosquito control.

## Background

Mosquitoes continue to be the major vectors of public health importance and few species of *Anopheles* genera transmit malaria. There are about 465 *Anopheles* species of which 70 species act as vectors [1]. In India, 58 species of *Anopheles* have been recorded of which six are primary vectors of malaria and four species act as secondary vectors playing significant role in disease transmission in some locations [2]. In 2019, a total of 227 million global malaria cases were recorded which rose to 241 million cases in 2020 [3]. Among these, about 95% cases are reported from the World Health Organization (WHO) African Region while the South-East Asian region recorded 2% of the global malaria burden. The South-East Asian region documented a significant reduction (78%) in malaria cases from 23 million in 2020 to about 5 million in 2020. Among various countries in the region, India documented 83% of the cases and 82% of the deaths [3].

The goal of malaria elimination by 2030 was set by the WHO. India has also committed to achieve elimination by 2027 [4]. In the absence of effective malaria vaccine, the only option to prevent the disease is the use of effective vector control interventions and chemotherapy. The vector control options mainly include adulticidal interventions of pyrethroid-based indoor residual sprays (IRS) and insecticide-treated nets (ITNs). Though, use of these interventions has decreased the disease burden, the major challenge experienced by disease control programmes in India is insecticide resistance in vectors [5–7].

Attractive Toxic Sugar Bait (ATSB) is one of the new control strategies based on the lure and kill approach which minimises the use of chemical insecticides. The approach is based on the fact that mosquitoes feed on plant sugars derived from floral sources (nectars, honeydew, and fruits juices) to meet their energy demands. These sources are located by the mosquitoes through various visual and olfaction cues. Some olfactory receptors, such as odorant receptors (ORs), are responsive to specific odours and need the obligate co-receptor for odour recognition. While, others, ionotropic receptors (IRs) recognize several classes of chemical compounds, including amines, aldehydes, ketones, and carboxylic acids [8]. Hence, the ATSB formulation, used as a bait, comprises a plant-derived olfaction stimulant combined with sugar and an insecticide.

ATSBs have been formulated using boric acid [9], dinotefuran [10], pyriproxyfen [11], spinosad [12], sodium ascorbate [13] and microencapsulated garlic oil [14]. In addition, the ATSB formulations have also been prepared with pyrethroid insecticides, which can enter the mosquitoes through cuticle while feeding [15]. Earlier, the toxic sugar baits (TSBs) containing active ingredients belonging to five chemical classes; macrocyclic lactones (2.46% spinosad, 0.1% ivermectin); neonicotinoids (0.5% imidacloprid, 21.6% thiamethoxam); pyrethroids (36.8% permethrin, 11.8% cyfluthrin, 7.9% bifenthrin, 4.75% deltamethrin); phenylpyrazole (9.1% fipronil) and pyrrole (21.45% chlorfenapyr) have been assessed against *Culex quinquefasciatus*, *Anopheles quadrimaculatus*, and *Aedes taeniorhynchus* [16]. Different efficacies of each TSB for different species of mosquitoes proposed the use of a blend of multiple active ingredients in the formulation for more efficient results.

Despite the fact that ATSB approach is simple to use, safer, successful and cost-effective than chemical insecticide-based interventions, and can be used indoors as well as outdoors, this vector control approach needs to be developed further and standardized for large scale use in the fields. The present study formulated an ATSB against *An*.* stephensi* by combining 10% sucrose solution as a source of sugar, fermented fruit juice as an attractant and deltamethrin as a toxicant. Initially nine ASB’s (without toxicant) were prepared with nine fruit juices and sucrose solution [17] and screened against a laboratory strain and a field-collected strain of *An*.* stephensi*. The most effective ASB was then combined with ten different concentrations of deltamethrin to prepare ten ATSB formulations which were assayed against two strains of *An*.* stephensi* [the NIMR strain (Sonepat) and the AND strain (GVD-Delhi)] to identify the most effective ATSB formulation.

## Methods

### Rearing and maintenance of mosquito in insectary

NIMR strain of *An*.* stephensi*—Collected from Sonepat, Haryana (29.0523°N, 76.9182°E), India in 1996 and established at NIMR (National Institute of Malaria Research), India without insecticide selection pressure (LT_50_ to deltamethrin = 3.37 min).

AND strain of *An*.* Stephensi*—Collected from the Govindpuri (GVD), Southeast Delhi (28.534°N, 77.265°E), India in October, 2021 and kept at Acharya Narendra Dev College (AND), India under sustained deltamethrin selection at adult stage (LT_50_ to deltamethrin = 4.36 min).

These two strains of *An*.* stephensi* were maintained in the Insect Pest and Vector Control Laboratory, Acharya Narendra Dev College, University of Delhi, India under controlled conditions of temperature (27 ± 2 °C), relative humidity (80 ± 10%) and Light–Dark photoperiod (14:10) using standard rearing methods [17].

Adult mosquitoes were reared in muslin cloth cages and fed on sucrose solution (10%) soaked in a cotton swab. Female mosquitoes were provided with blood meal on alternate days using albino mice. Eggs were collected in an ovicup kept inside the cage and hatched in an enamel/plastic tray filled with dechlorinated water. The larvae were fed on finely powdered dog biscuits and yeast (3:2 w/w). The pupae were collected in a plastic bowl and kept in the cage for adult emergence. The studies were conducted on the 2–3 day old, and non-blood fed healthy adults starved for duration optimized as 24 h for preliminary bioassays.

### Identification of effective attractive sugar baits (ASBs)

#### Preparation of ASB

A total of nine ASBs were prepared by mixing 10% sucrose solution (w/v) and the fermented fruit juices (1:1 v/v). The juices were prepared from nine locally available fruits; *Ananas comosus* (pineapple), *Carica papaya* (papaya), *Citrus limetta* (sweet lemon), *Citrus sinensis* (orange), *Citrullus lanatus* (watermelon), *Cucumis melo* (muskmelon), *Mangifera indica* (mango), *Prunus domestica* (plum) and *Psidium guajava* (guava); using a mixer-grinder (Powermatic Plus, Sujata Appliances India). The extracted juices were stored in a closed reagent bottle and fermented for 48 h, at ambient temperature (27 ± 2 °C) of laboratory in order to enhance their odour. Each fermented juice was combined with 10% sucrose solution (w/v) in 1:1 ratio to formulate the ASBs. Control assays were run with only sucrose solution (10% w/v).

### Cage bioassays to select efficient ASB

#### Pre-screening bioassay

**Fig. 1:**
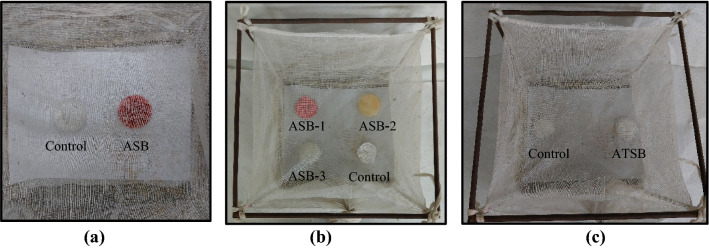
Cage bioassay with *Anopheles stephensi* adults (n = 50; 25 males and 25 females). **a** Pre-screening cage setup with one ASB and a control (10% sucrose solution) bait placed at two sides. **b** Screening cage setup with three ASBs (eg. ASB-1, 2, 3) and a control (10% sucrose solution) bait placed in four corners. **c** Screening cage setup with ATSB formulation (Guava juice-ASB + deltamethrin) and a control (10% sucrose solution) bait placed at two sides

A total of eighteen cotton discs (0.5 g), nine experimental and nine controls, were taken. Experimental discs were saturated with 5 mL of an ASB while control discs were soaked in 5 mL of 10% sucrose solution. One experimental and one control disc kept in separate Petri plates were placed at the two sides of an adult cage (45 × 40 × 40 cm) (Fig. 1a). A total of 50 adults (2–3 days old) of *An*.* stephensi* (25 females and 25 males) were released into the cage. The landings of the mosquitoes on each disc were scored for an hour at every 10 min interval, or till the landings ceased. The discs were interchanged after every score to prevent the position effect. The assay was carried out in four replicates with each ASB for both Sonepat (NIMR) and GVD-Delhi strains of *An*.* stephensi*. Three most effective ASBs were identified for screening bioassay.

#### Screening bioassay

Three ASBs cotton discs, one soaked in an ASB among three identified in pre-screening assay and two soaked in randomly selected ASBs from the remaining six ASBs, were placed at the three corners of a screened cage, while the control disc was placed at the fourth corner (Fig. 1b). The random combination of ASBs used in three cages were as follows.

- Cage 1: Mango juice-ASB, muskmelon juice-ASB and watermelon juice-ASB.
- Cage 2: Orange juice-ASB, papaya juice-ASB and plum juice-ASB.
- Cage 3: Guava juice-ASB, pineapple juice-ASB and sweet lemon juice-ASB.

The protocol similar to pre-screening assay was followed. The average landing counts on each ASB in each cage were recorded.

#### Statistical analysis

The standard error of mean (SEM) of landings on each ASB was calculated. Data was analysed by one-way ANOVA followed by Tukey’s all pairwise multiple comparison test with *p* value < 0.05 considered as the significant. In each cage, the most effective ASB attracting significantly higher number of adult mosquitoes in comparison to other two ASBs (*p* < 0.05) was selected.

#### Post-screening bioassay

Four cotton discs, three soaked in identified ASBs and fourth in control solution were placed at the four corners of a cage. The average landing counts of mosquitoes on each disc were scored as earlier and analysed statistically. The attraction potential of the ASBs was compared by calculating an attraction index using the following formula (Eq. 1).

1 $$Attraction\,Index = \frac{Mean\,number\,of\,mosquitoes\,attracted\,to\,the\,bait}{{Mean\,number\,of\,mosquitoes\,attracted\,to\,the\,control}}$$

Percentage attractancy of each bait was calculated using the Eq. 2.

2 $$Percentage\,attractancy = \frac{Number\,of\,landings\,of\,mosquito\,on\,a\,particular\,attractant}{{Total\,number\,of\,landings\,on\,all\,the\,attractant}} \times 100.$$

### Formulation of attractive toxic sugar bait (ATSB)

#### Preparation of ATSB

The ASB with maximum attractant potential for *An*.* stephensi*, selected on the basis of cage bioassays, was used to prepare ATSB solution. The pyrethroid insecticide, deltamethrin (toxic component), was added to the ASB in 1:9 ratio. A total of ten ATSB solutions were prepared with 1 mL of 0.0015625–0.8% deltamethrin mixed with 9 mL of ASB (0*.*01562*–*8.0 mg deltamethrin/10 mL ATSB solution).

#### Cage bioassay with ATSBs

The bioassay with different ATSB formulations was carried out in separate screened cloth cages (1, 2 and 3). In each cage, an experimental cotton disc soaked in 5 mL of an ATSB and a control cotton disc with 5 mL of 10% sucrose solution were placed on the two sides (Fig. 1c). A total of 50 adults (2–3 days old) of *An*.* stephensi* (25 females and 25 males) were released into the cage. The total number of mosquitoes died/knocked down were scored after 24 h and 48 h.

#### Statistical analysis

The average number of mosquitoes, dead/knocked down, on each ATSB was analysed statistically by one-way ANOVA and Tukey’s all pairwise multiple comparison test using PASW (SPSS) software 19.0 program. The *p* value < 0.05 was considered as the significant value. The bioassays with more than 20% mortality in controls were rejected and repeated. The mortality values on ATSB were corrected using the Abbott’s formula [18] given in Eq. 3, if the mortality in control ranged between 5 and 20%

3 $${\text{Corrected}}\,{\text{mortality}}\,(\% ) = \frac{T - C}{{100 - C}} \times 100$$

where T is the percent mortality on ATSB and C is the percent mortality on controls.

## Results

### Cage bioassays with ASB’s

The number of mosquitoes of the NIMR strain and the AND strain of *An. stephensi* attracted towards an ASB along with their respective control in pre-screening bioassays, is presented in Table 1. The number of NIMR adults landed on different ASBs was scored in the range of 3.5–18.25, while landing counts of the AND strain mosquitoes ranged from 5.0 to 19.50. The guava juice-ASB displayed highest attracting potential followed by plum juice and mango juice-ASB’s towards both the strains of *An*.* stephensi*. The total number of mosquitoes attracted by the remaining six ASBs was less than the mosquitoes landed on the corresponding control bait with the pineapple juice-ASB showing the least attracting potential (Table 1).

**Table 1:** Number of adults *Anopheles stephensi* of Sonepat (NIMR strain) and GVD-Delhi (AND strain) attracted towards different attractive sugar baits (ASB’s) in pre-screening cage bioassays

S. no.	ASB	*Anopheles stephensi*			
		Sonepat (NIMR strain)		GVD-Delhi (AND strain)	
		No. of mosquitoes landed on the bait ± SEM	Control	No. of mosquitoes landed on the bait ± SEM	Control
1	Water melon	6.00 ± 0.408 a *	10.75 ± 0.478 a #	7.50 ± 0.288 a *	11.25 ± 0.478 a #
2	Muskmelon	5.75 ± 0.75 a *	11 ± 0.408 a #	6.75 ± 0.75 ab *	11.5 ± 0.288 a #
3	Orange	7.25 ± 0.629 a *	9.75 ± 0.250 b *	8.00 ± 0.408 a *	10.50 ± 0.288 a *
4	Sweet lemon	8.00 ± 0.577 a *	12.25 ± 0.629 a #	9.00 ± 0.408 a *	12.75 ± 1.108 a #
5	Papaya	7.00 ± 0.408 a *	8.25 ± 0.250 c #	8.50 ± 0.645 a *	9.00 ± 0.408 b #
6	Mango	10.75 ± 0.25 b *	9.0 ± 0.408 bc #	11.50 ± 0.645 c *	10.0 ± 0.408 b #
7	Plum	14.25 ± 0.629 c *	8.25 ± 0.478 c #	15.00 ± 0.707 d *	8.75 ± 0.629 b #
8	Pineapple	3.5 ± 0.500 d *	9.25 ± 0.478 bc #	5.00 ± 0.408 b *	9.50 ± 0.645 b #
9	Guava	18.25 ± 0.478 e *	10.75 ± 0.478 a #	19.50 ± 0.645 e *	11.00 ± 0.408 a #

*Four replicates each with n = 50, 25 males and 25 females (1 h @ intervals of 10 min), total n = 200

Values in the table represent number of mosquito landings; ASBs with different letters (column-wise) and different symbols (row-wise) are significantly different (*p* < 0.05) computed by one-way ANOVA followed by Tukey’s all pair wise multiple comparison test

**Fig. 2:**
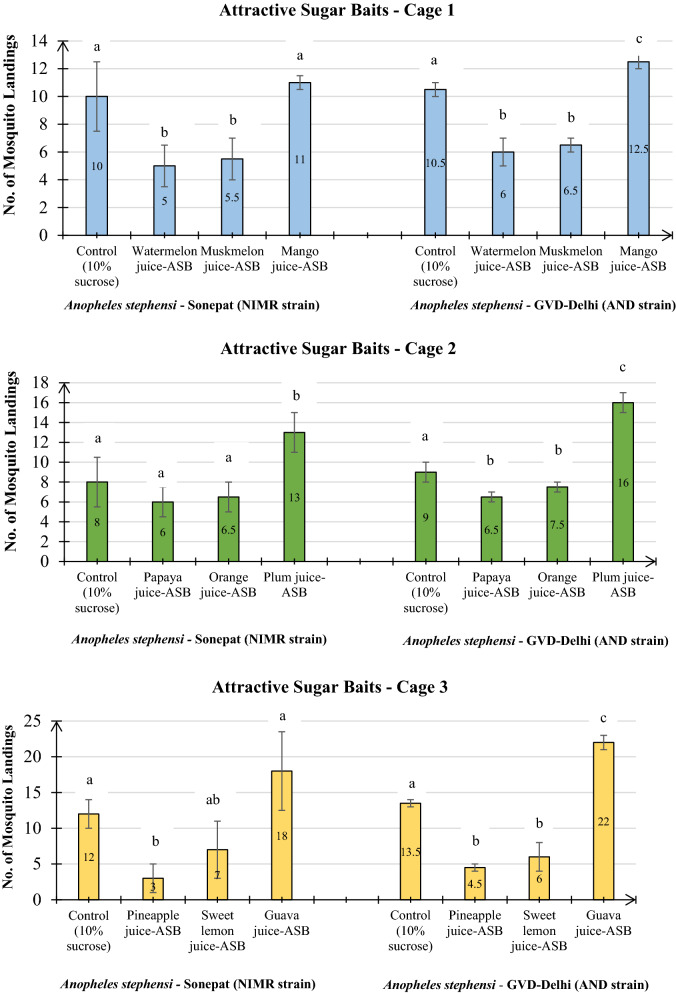
Screening assay showing number of landings in Sonepat (NIMR strain) and GVD-Delhi (AND strain) of *Anopheles stephensi* on three ASBs placed along with control in one cage. *Four replicates each with n = 50, 25 males and 25 females (1 h @ intervals of 10 min). Values in the bars represent number of mosquito landings; ASBs with different letters indicated on the bars are significantly different (*p* < 0.05) computed by one-way ANOVA followed by Tukey’s all pairwise multiple comparison test

The screening assays with groups of three ASBs and a control showed 6–36% landings on the ASBs and 16–24% landings on the control bait using the NIMR strain; while with the AND strain 9–44% landings were scored on ASBs as against 18–27% on the control bait (Table 2). The highest attractancy (18 landings) was recorded by guava juice-ASB among all the nine ASBs followed by plum (13) and mango juice-ASB (11) against the NIMR strain (Fig. 2). Relatively smaller number of mosquitoes landed on pineapple (3), watermelon (5), muskmelon (5.5), papaya (6), orange (6.6) and sweet lemon (7) ASBs (*p* > 0.05). The landings on control discs (10% sucrose solution) were noted in the range of 8–12 in the three cages. Likewise, screening bioassays with the AND strain showed highest landings on guava juice-ASB (22) followed by plum (16) and mango juice-ASBs (12.5), while the number of landings were lowest on pineapple juice-ASB (4.5) and moderate on sweet lemon and watermelon (6), papaya and muskmelon (6.5), and orange (7.5) ASBs (*p* < 0.05) (Fig. 2).

**Table 2:** Percent *Anopheles stephensi* mosquitoes of Sonepat (NIMR strain) and GVD-Delhi (AND strain) attracted towards different Attractive Sugar Baits (ASB’s) in screening and post-screening assays

ASBs	% *Anopheles stephensi* adults landed on the bait	
	Sonepat (NIMR strain) (n = 50) (%)	GVD-Delhi (AND strain) (n = 50) (%)
Screening Cage-1		
Control	20.0	21.0
Watermelon juice-ASB	10.0	12.0
Muskmelon juice-ASB	11.0	13.0
Mango juice-ASB	22.0	25.0
Screening Cage-2		
Control	16.0	18.0
Papaya juice-ASB	12.0	13.0
Orange juice-ASB	13.0	15.0
Plum juice-ASB	26.0	32.0
Screening Cage-3		
Control	24.0	27.0
Pineapple juice-ASB	06.0	9.0
Sweet lemon juice-ASB	14.0	12.0
Guava juice-ASB	36.0	44.0
Post-screening		
Control (10% sucrose)	20.0	24.0
Mango juice-ASB	24.0	29.0
Plum juice-ASB	24.0	31.0
Guava juice-ASB	30.0	33.0

*Four replicates each with n = 50, 25 males and 25 females (1 h @ intervals of 10 min), total n = 200

**Fig. 3:**
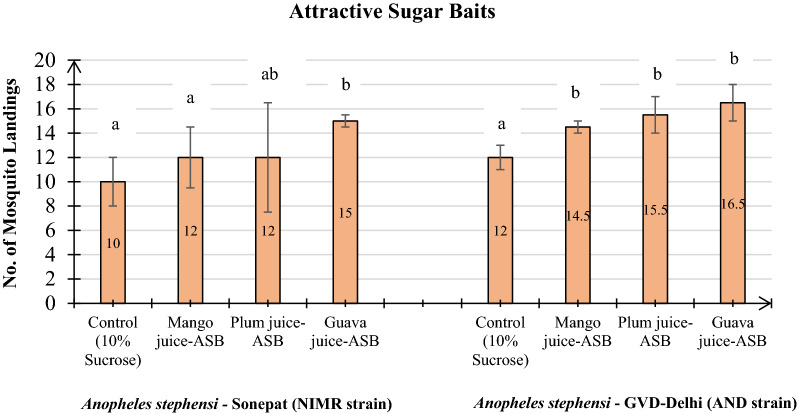
Post-screening assay showing number of landings in Sonepat (NIMR strain) and GVD-Delhi strains of *An. stephensi* on three most efficient ASBs placed along with control in one cage. *Four replicates each with n = 50, 25 males and 25 females (1 h @ intervals of 10 min). Values in the bars represent number of mosquito landings; ASBs with different letters indicated on the bars are significantly different (*p* < 0.05) computed by one-way ANOVA followed by Tukey’s all pair wise multiple comparison test

The post-screening assays with the three identified effective ASBs; Guava, Plum and Mango juice-ASBs; along with a control showed maximum attraction potential of guava juice-ASB (16.5, 15) for both the NIMR strain and the AND strain of *An*.* stephensi* (*p* < 0.05). The attractancy potential of plum juice and mango juice-ASB for the AND strain was 15.5 and 14.5, respectively, while attractancy potential of these two ASBs (12) was same for the NIMR strain (Fig. 3).

The relative attractant potential (mean number of mosquitoes attracted to the baits/mean number of mosquitoes attracted to the control) of the three ASBs assayed as compared to the control in post-screening tests showed highest relative attractancy of guava juice-ASB towards both the strains of *An. stephensi* recorded as 1.50 for Sonepat (NIMR) strain and 1.37 for GVD-Delhi (AND strain) followed by plum juice-ASB (1.29) against GVD-Delhi (AND strain). The lowest relative attractancy was showed by plum juice-ASB (1.20) for Sonepat (NIMR strain) and Mango juice-ASB (1.20) against both Sonepat (NIMR strain) and GVD-Delhi (AND train) of *An. stephensi* (Table 3)*.*

**Table 3:** Relative attractant efficacy* of three ASB’s for Sonepat (NIMR strain) and GVD-Delhi (AND strain) of *Anopheles stephensi* with respect to the control in post-screening assays

*Anopheles stephensi*	Fruit juice in ASB formulation			
	Control	Mango	Plum	Guava
Sonepat (NIMR strain)	1.00	1.20	1.20	1.50
GVD-Delhi (AND strain)	1.00	1.20	1.29	1.37

*Relative attractant efficacy: mean number of mosquitoes attracted to the baits/mean number of mosquitoes attracted to the control

### Cage bioassays with ATSB

On the basis of above results, the guava juice-ASB, with maximum attractancy potential for mosquitoes, was used to prepare ATSB’s with different dosages of deltamethrin. The number of dead mosquitoes recorded on different ATSB’s was positively correlated with the concentration of deltamethrin in ATSB. After 24 h holding, the % mortality with ATSB containing 0.0015625–0.8% deltamethrin recorded was 5.10–97.96% against Sonepat (NIMR strain) and 6.12–96.91% against GVD-Delhi (AND strain). The lowest percent mortality in the adults of Sonepat (NIMR strain) strain and GVD-Delhi (AND strain) was observed as 5.10% and 6.12%, respectively, with ATSB containing 0.0015625% deltamethrin. With the rising concentration of deltamethrin in the ATSB formulation, the adult mortality also increased in both the strains. The total mortality recorded with 0.03125% and 0.0625% deltamethrin-ATSB were 8.67 and 10.93 (*p* < 0.05) in Sonepat (NIMR strain) in comparison to just 3.61 and 7.14 (*p* < 0.05) in GVD-Delhi (AND strain) after 24 h (Table 4). The ATSB with 0.0125%, 0.025% and 0.05% deltamethrin caused 14.73–18.04 mortality in Sonepat (NIMR strain) while comparatively less mortality, ranging from 8.38 to 17.71, was scored in GVD-Delhi (AND strain) (*p* > 0.05) (Table 4). The respective mortality in these two strains increased by 15-fold and 12-fold with ATSB containing 0.4% deltamethrin and further by 19-fold and 16-fold with 0.8% deltamethrin-ATSB (Table 4).

**Table 4:** Number of *Anopheles stephensi* adults of Sonepat (NIMR strain) and GVD-Delhi (AND strain) attracted and killed towards ATSB formulation during ATSB cage bioassays

ATSB (guava juice-ASB + deltamethrin %)	% Mortality of *Anopheles stephensi* adults on the bait	
	Sonepat (NIMR strain)	GVD-Delhi (AND strain)
	No. of mosquitoes died on the bait ± SEM	No. of mosquitoes died on the bait ± SEM
0.8	48.97 ± 0.00 a (97.96%)**	48.45 ± 0.00 a (96.91%)
0.4	38.74 ± 3.50 b (77.49%)	36.84 ± 2.00 b (73.68%)
0.2	32.98 ± 2.00 c (65.98%)	34.38 ± 1.00 b (68.75%)
0.1	23.43 ± 1.50 d (46.88%)	28.87 ± 1.00 c (57.73%)
0.05	18.04 ± 0.50 e (36.08%)	17.71 ± 3.00 d (35.42%)
0.025	16.23 ± 2.00 e (32.46%)	13.16 ± 1.50 d (26.32%)
0.0125	14.73 ± 1.00 e (29.47%)	8.38 ± 0.50 d (16.75%)
0.00625	10.93 ± 0.50 f (21.88%)	7.14 ± 1.00 e (14.29%)
0.003125	8.67 ± 1.50 g (17.35%)	3.61 ± 1.50 ef (7.22%)
0.0015625	2.55 ± 1.50 h (5.10%)	3.06 ± 1.00 f (6.12%)

*Four replicates each with n = 50, 25 males and 25 females (24 h), total n = 200

**% Mortality corrected using Abbott’s formula (1925); values in the table represent number of mosquitoes died; ATSBs with different letters (column-wise) are significantly different (*p* < 0.05) computed by one-way ANOVA followed by Tukey’s all pair wise multiple comparison test

The ATSB cage bioassay against Sonepat (NIMR strain) resulted in calculated LC_30,_ LC_50_ and LC_90_ values of 0.17 mg deltamethrin/10 mL, 0.61 mg deltamethrin/10 mL and 13.84 mg deltamethrin/10 mL ATSB, respectively, while the corresponding values recorded against GVD-Delhi (AND strain) of *An. stephensi* were 0.25 mg deltamethrin/10 mL, 0.73 mg deltamethrin/10 mL and 10.22 mg deltamethrin/10 mL ATSB, respectively (Table 5).

**Table 5:** Lethal concentrations of deltamethrin in ATSB formulation assayed against Sonepat (NIMR strain) and GVD-Delhi (AND strain) of *Anopheles stephensi*

Strain	Lethal concentrations (LCs) of ATSB (%)						
	LC_30_	LC_50_	LC_90_	S.E	χ^2^	DF	R.C
Sonepat (NIMR strain)	0.017^#^ (0.012–0.023)*	0.061 (0.045–0.084)	1.384 (0.776–3.032)	0.082	14.708	8	0.945
GVD-Delhi (AND strain)	0.025 (0.018–0.033)	0.073 (0.056–0.097)	1.022 (0.634–1.915)	0.089	8.632	8	1.117

*Values in parentheses indicate 95% confidential limits; SE: Standard Error; χ^2^: Chi-square; DF: Degree of Freedom; RC: Regression Coefficient

^#^Deltamethrin dosages in mg/10 mL of ATSB: LC_30_ = 0.17 mg/10 mL, LC_50_ = 0.61 mg/10 mL, LC_90_ = 13.84 mg/10 mL of ATSB [Sonepat (NIMR strain)]; LC_30_ = 0.25 mg/10 mL, LC_50_ = 0.73 mg/10 mL, LC_90_ = 10.22 mg/10 mL of ATSB [GVD-Delhi (AND strain)]

## Discussion

The ATSB, a blended formulation of fruit juice/flower nectar, a toxin, and sugar solution is a recently developed innovative strategy against mosquitoes. The ATSB approach is considered an effective, technically simple and low-cost solution to avoid the issues and concerns associated with contact insecticides [19] as the formulated bait works by competing with naturally accessible sources of plant sugar, the food and energy source for the mosquitoes.

The toxic sugar baits (TSB) comprising a combination of sugar and toxicant (malathion) have been used earlier to control *Aedes aegypti* [20]. The potential of TSBs containing various other insecticides (bifenthrin, cyfluthrin, deltamethrin, permethrin) have been tested against different mosquito species; *Cx. quinquefasciatus*, *An. quadrimaculatus*, *Ae. taeniorhynchus*, *Culex nigripalpus*, and *Aedes albopictus* [16, 21, 22]. Though laboratory trials with these TSBs were found effective, the field trials could not lure mosquitoes and give efficient results due to the presence of natural sugar sources in the environment. Therefore, the formulation of ATSBs was recommended with adding fruit juices, flower nectar, or insect honeydew [23, 24].

The present study identified an effective attractant, optimized the concentration of toxicant and formulated an effective ATSB against malaria vector, *An. stephensi*. The efficacy of ATSB was evaluated against the NIMR strain and the AND strain of *An. stephensi*. As the attractant is a significant component in ATSB in order to lure the adult mosquitoes on the bait, initially nine ASBs were prepared with different fruit juices and evaluated for their attraction potential against the two strains. The ASBs formulated with guava juice, plum juice and mango juice exhibited significantly higher attractancy against both the strains in comparison to the control (*p* < 0.05) and rest of the ASBs with other fruit juices. The assays ascertaining the relative attraction potential of the juices revealed the highest attractancy of guava juice-ASB in comparison to the rest of fruit juice ASBs (*p* < 0.05) for both the NIMR strain and the AND strain. The other two ASBs found effective were plum juice-ASB and mango juice-ASB. Similar results were obtained in earlier experiments when nine ASBs were tested against two laboratory strains (the AND strain of *Ae. aegypti*, and the DL10 strain of *Ae. aegypti*) and two field strains of *Ae. aegypti* (SHD-Delhi and GVD-Delhi)*.* Against all the four strains, the guava juice-ASB exhibited the highest attractant potential followed by plum and mango juice-ASBs. However, the guava juice-ASB possessed 1.22 to 1.4-fold higher attraction potential for *An. stephensi* strains in comparison to *Ae. aegypti* [17]. The optimization of toxin dosage to be added in ATSB formulations against these *Ae. aegypti* strains and cage as well as field bioassays is in progress.

Similar studies were held in Bagamoyo, Tanzania to assess the attraction potential of seven ASBs on *Anopheles arabiensis*, banana (*Muso*), guava (*Psidium guajava*), mango (*Mangifera indica*), orange (*Citrus sinensis*), papaya (*Carica papaya*), tomato (*Solanum lycopersicum*) and watermelon (*Citrullus lanatus*) pulps, and showed significant attractant potential of orange juice-ASB > tomato juice-ASB > guava juice-ASB [25]. In Mali, Muller et al*.* [23] evaluated the attractancy potential of locally available 26 types of fruits/seedpods and 26 different flowering plants for malaria vector, *Anopheles gambiae* and demonstrated significant attraction potential of the 6 species of fruits and 9 species of flowering plants with *Acacia macrostachya* identified as the most attractive flowering plant, while guava and muskmelon (*Cucumis melo*) as the most attractive fruits.

The current study formulated an ATSB with Guava juice-ASB and the toxic component, deltamethrin. Nine ATSBs were prepared containing different concentrations of deltamethrin and were tested against both the strains of *An. stephensi* to determine their efficacy. The assays revealed a dose-dependent effect of ATSBs resulting in higher mortality of *An. stephensi* adults with the increasing deltamethrin concentration in the ATSB, the 0.8% deltamethrin-ATSB registered 97.96% mortality in the NIMR strain and 96.91% in the AND strain of *An. stephensi*. The LC_50_ values recorded with ATSBs were 0.061% and 0.073% against the NIMR strain and the AND strain of *An. stephensi*, respectively, after 24 h post introduction of different dosages of ATSB.

Similar assays with various guava juice-ASBs combined with 0.5% chlorfenapyr, 2% boric acid, or 1% tolfenpyrad resulted in > 90% mortality in pyrethroid-susceptible population of *An. gambiae*, as well as pyrethroid-resistant population of *An. arabiensis* and *Cx. quinquefasciatus*. However, the hut trials with these ATSBs could cause just 41–48% mortality in *An. arabiensis* and 36–43% mortality in *Cx. quinquefasciatus* [26]. Likewise, ATSB formulated with mango juice, guava juice, brown sugar and boric acid resulted in 100% mortality of *Ae. albopictus* in laboratory trials, while 95% and 58% mortality under semi-field and field trials, respectively [27].

The bioassay with ATSB containing guava juice-ASB and 0.2–2% boric acid or 0.05–0.5% chlorfenapyr against *An. gambiae* showed 100% mortality at 2% boric acid and 0.5% chlorfenapyr against both the susceptible (*Kisumu*) and resistant (*M’bé*) strains [28]*.* In Mali, ATSB containing guava and honey melon juice (1:1), sugar and boric acid caused 83.78% population reduction of *An. gambiae* within a month after its application [23], while in Israel, same formulation reduced nearly 90% *An. gambiae* population just after 1 week [29]. Another study in Israel held with ATSB (75% juice of *Opuntia ficus-indica*, 5% wine, 20% brown sugar, 1% BaitStab™ and 1% boric acid) reduced daily survival rates of *Anopheles* species [19].

Current study investigated a contact insecticide, deltamethrin, in the ATSB, against *An. stephensi*, which was found effective in controlling mosquito population in the field. Till date, limited studies have been conducted with contact insecticides-ATSBs. Most of the ATSB studies have been carried out with baits containing oral toxicants, such as dinotefuran, spinosad, chlorfenapyr and boric acid. The efficacy of three ATSBs, two containing oral toxicants—1.0% boric acid, 0.5% dinotefuran; and one with contact toxicant—0.1% deltamethrin, was assessed against both susceptible and deltamethrin-resistant strains of *Cx. quinquefasciatus* [30]*.* The results showed higher efficacy of all the ATSBs against resistant populations than the susceptible ones, probably due to the lower survival fitness of resistant population in the fields. In comparison to ATSBs containing boric acid and dinotefuran, the efficacy of deltamethrin-containing ATSB was lower against the deltamethrin-resistant population. It was suggested that the resistant population was more susceptible to the boric acid and dinotefuran than deltamethrin because of the different mechanisms of action and absence of cross-resistance to deltamethrin [30].

Presently, malaria vector management is reliant on the pyrethroids used in IRS and LLINs which has resulted in the development of resistance in mosquitoes [5]. Evidences have shown that pyrethroid-resistant adult mosquitoes have developed cross-resistance to other insecticides with same mechanism of action because of metabolic detoxification or insensitivity of the target site [31]. Such studies indicate that mosquitoes have ability to develop resistance to ATSBs because of use of toxicants with same mechanism of action as that of pyrethroids. However, no such studies have been carried out till date. It is believed and recommended that rotation of toxicants with different mechanisms of action in ATSBs, can not only mitigate the problems associated with the additional pressure selecting for the development of pyrethroid resistance but also can cause reversion of resistance in the field as suggested for other interventions.

ATSB methods have been suggested as effective tools for mosquito management in the fields. However, very few reports have assessed the environmental concerns associated with their use and thus needs to be investigated extensively for their effects on the non-targets. The available reports have suggested their safer use in non-flowering areas in comparison to the flowering areas. Eugenol containing-ATSB sprayed to control *Ae. albopictus* impacted 5.5% of the non-target insects investigated, when applied in the flowering vegetation while only 0.6% insects fed on the ATSB in non-flowering conditions indicating safety of the bait in the field [32]. Likewise, garlic oil-containing ATSB against *Anopheles sergentii* population showed minimal effects on non-target insects when applied to foliage of non-flowering plants as compared to the flowering plants [33]. Reports regarding non-target impact of ATSB-Pyrethroids are still lacking. However, considering their known safety against non-target insects, however, make them plausible tools for mosquito control in the field.

Based on the results obtained in current laboratory study, an extensive work is proposed to be carried out to setup trials for the assessment of the developed guava + deltamethrin ATSB formulation in the field conditions to assess the feasibility of use of this approach in mosquito management.

## Conclusions

The current study prepared an ATSB formulation against the NIMR strain and the AND strain of *An. stephensi.* Pre-screening and screening bioassays with nine fruits juices revealed the significant attractant potential of guava juice-ASB, plum juice-ASB and mango juice-ASB in that order. Further studies ascertained the maximum efficacy of guava juice-ASB, which was then mixed with different concentrations of deltamethrin to optimize the dosage of toxin. The ATSB formulation with 0.8% deltamethrin caused highest (97.96–96.91%) mortality against both the NIMR and the AND strains of *An. stephensi* within 24 h of treatment. Rest of the formulations caused 5.10–77.49% mortality though all the formulations led to complete adult mortality after 48 h. These studies showed the efficacy of formulated ATSB against *An. stephensi* irrespective of their pyrethroid susceptibility level. It recommends that application of ATSB outdoors could be used as a probable tool for possible impact on outdoor prevalence of vector and disease transmission.

## Data Availability

All data generated or analysed during this study are included in this article.

## Abbreviations

AND: Acharya Narendra Dev
ASB: Attractive sugar bait
ATSB: Attractive toxic sugar bait
GVD: Govindpuri
IRS: Indoor residual sprays
IRs: Ionotropic receptors
ITNs: Insecticide-treated nets
LC: Lethal concentration
LT: Lethal time
NIMR: National Institute of Malaria Research
ORs: Odorant receptors
TSBs: Toxic sugar baits
WHO: World Health Organization
